# Comparative venomic profiles of three spiders of the genus *Phoneutria*

**DOI:** 10.1590/1678-9199-JVATITD-2021-0042

**Published:** 2022-02-09

**Authors:** Frederico Francisco Fernandes, Juliana Rodrigues Moraes, Jaqueline Leal dos Santos, Thiago Geraldo Soares, Vitor José Pinto Gouveia, Alessandra C. S. Matavel, William de Castro Borges, Marta do Nascimento Cordeiro, Suely Gomes Figueiredo, Márcia Helena Borges

**Affiliations:** 1 Laboratory of Proteomics and Arachnids, Research and Development Board, Ezequiel Dias Foundation, Belo Horizonte, MG, Brazil.; 2 Laboratory of Biotechnology and Health, Research and Development Board, Ezequiel Dias Foundation, Belo Horizonte, MG, Brazil.; 3 Laboratory of Enzymology and Proteomics, Department of Biological Sciences, Federal University of Ouro Preto, Ouro Preto, MG, Brazil.; 4 Laboratory of Protein Chemistry, Department of Physiological Sciences, Federal University of Espírito Santo, Vitória, ES, Brazil.

**Keywords:** Spider venom, Phoneutria, Protein profile, Toxins, Ion channels

## Abstract

**Background:**

Spider venoms induce different physio-pharmacological effects by binding with high affinity on molecular targets, therefore being of biotechnological interest. Some of these toxins, acting on different types of ion channels, have been identified in the venom of spiders of the genus *Phoneutria*, mainly from *P. nigriventer*. In spite of the pharmaceutical potential demonstrated by *P. nigriventer* toxins, there is limited information on molecules from venoms of the same genus, as their toxins remain poorly characterized. Understanding this diversity and clarifying the differences in the mechanisms of action of spider toxins is of great importance for establishing their true biotechnological potential. This prompted us to compare three different venoms of the *Phoneutria* genus: *P. nigriventer* (Pn-V), *P. eickstedtae* (Pe-V) and *P. pertyi* (Pp-V).

**Methods:**

Biochemical and functional comparison of the venoms were carried out by SDS-PAGE, HPLC, mass spectrometry, enzymatic activities and electrophysiological assays (whole-cell patch clamp).

**Results:**

The employed approach revealed that all three venoms had an overall similarity in their components, with only minor differences. The presence of a high number of similar proteins was evident, particularly toxins in the mass range of ~6.0 kDa. Hyaluronidase and proteolytic activities were detected in all venoms, in addition to isoforms of the toxins Tx1 and Tx2-6. All Tx1 isoforms blocked Nav1.6 ion currents, with slight differences.

**Conclusion:**

Our findings showed that Pn-V, Pe-V and Pp-V are highly similar concerning protein composition and enzymatic activities, containing isoforms of the same toxins sharing high sequence homology, with minor modifications. However, these structural and functional variations are very important for venom diversity. In addition, our findings will contribute to the comprehension of the molecular diversity of the venoms of the other species from *Phoneutria* genus, exposing their biotechnological potential as a source for searching for new active molecules.

## Background

Among the components of spider venoms, a wide variety of toxins evolved to bind to specific targets of biological importance, such as ion channels, inducing biological effects that allow spiders to immobilize their prey in a very efficient way, using minor quantities of their toxins [1,2]. These venoms contributed to the enormous evolutionary success of spiders, providing the basis for their highly abundance on the planet. Spider venoms are being used for the discovery and development of new biologically active molecules for medical and biotechnological purposes, drug design, and application in agriculture or as pharmacological tools [3-8]. It is likely that many other applications will be discovered, because despite the large number of species already described, there is relatively limited knowledge concerning the composition of spider venoms.

The first studies on spider venoms started in the middle of the 20^th^ century and have advanced enormously up to the present time, suggesting the immeasurable biotechnological potential of these molecules [9]. In a recent review, Muttenthaler et al. [10] described that venom-derived peptides are also being used to drug development for the treatment of diseases, and they are providing templates for engineering of novel diagnostic agents and therapeutics.

Besides, the advances in high throughput methods, such as transcriptomics, and the use of mass spectrometry-based, large-scale proteomics allowed a faster identification and characterization of new molecules, with a minimum amount of the samples [11-13]. The findings have revealed the diversity of the venoms and are uncovering their biotechnological potential.

In Brazil, “Brazilian wandering spiders” or “banana-spiders” species of the genus *Phoneutria* Perty, 1833, are amongst the most explored and studied spider venoms, in particular *Phoneutria nigriventer*. *Phoneutria* sp. are synanthropic species and, therefore, they are responsible for the high number of human accidents caused by these arthropods. These accidents occur mainly in Brazil, but there are reports of cases in Central America and other nearby countries [14]. 

Species from the genus *Phoneutria*, family Ctenidae, suborder Araneomorphae, inhabit forests from Central America, South America, East of the Andes and North of Argentina. In Brazil, *P. nigriventer*, *P. keyserlingi*, *P. pertyi*, *P. bahiensis* and *P. eickstedtae* species are found in Atlantic Rainforest and forest fragments in the Cerrado. *P. nigriventer* species are the most widely distributed in Brazil, being found in the states of Minas Gerais, Goiás, Mato Grosso do Sul, Espírito Santo, Rio de Janeiro, São Paulo, Paraná, Santa Catarina and Rio Grande do Sul. *P. pertyi* species inhabit Atlantic rainforests, from the southeast of Bahia state, eastern parts of Minas Gerais, Espírito Santo and northwestern of Rio de Janeiro. *P. eickstedtae* species are found in fragments of Atlantic Rainforest in the Cerrado of Tocantins states, Goiás, Mato Grosso and Mato Grosso do Sul [15].

 Studies on *P. nigriventer* venom have been reported since the 1950s. They have shown the venom to be composed of molecules with diverse biological activities responsible for the envenomation effects [6,16,17]. *P. nigriventer* is one of the few spiders of medical relevance in the world, being responsible for the majority of human accidents with spiders, most of them in Brazil [18]. The accidents - referred to as phoneutrism - are characterized by local and systemic effects that include intense pain, edema, convulsions, agitation, nausea, lachrymation, excessive salivation, tremors, spastic paralysis, neurotoxicity, hypertension, cardiac arrhythmia, and penile erection (priapism) [18-20]. These effects are induced by a complex mixture of neurotoxins, short chain peptides (up to 2 kDa) and enzymes (such as hyaluronidase and peptidases) [21].

In previous studies, our research group purified and characterized four distinct families of neurotoxins from *P. nigriventer* venom, referred to as *Phoneutria* toxins (PhTx1, PhTx2, PhTx3 and PhTx4) [22]. PhTx refers to the fractions that encompass toxins, while the numbers (1, 2, 3 or 4) are derived from the first step of purification procedure. On the other hand, PnTx (for example PnTx1 or PnTx2-6) is the denomination of isolated toxins (peptides) from each specific fraction (for a review, see [17,21,22]). 

These are polypeptides with different primary structures (30-80 amino acid residues in length, MW 3.5-9.0 kDa) displaying different pharmacological properties [21]. These toxins comprise most of the venom compounds and interact with neuronal ion channels, modulating their activity, and with chemical receptors of the neuromuscular systems of mammals and insects, affecting neurotransmitter release [23]. Considering the ability of *Phoneutria* toxins to bind to ion channels, and the role these channels play in different types of biological events, including in some diseases (channelopathies), the biochemical characterization of its components is of major relevance. Previous investigations were focused on the biotechnological potential of these toxins [24], as models of therapeutic molecules [6], for cell permeability studies [25,26], or for the development of drugs that pass the blood-brain barrier [26-28].

Some *Phoneutria* toxins have been widely explored. Apart from the family PhTx1, from which only one toxin was identified (initially termed “Tx1” and lately PnTx1), the other families - PhTx2, PhTx3 and PhTx4 - have been described as collections of isotoxins (named PnTx2, PnTx3 and PnTx4) that share sequences with high identity and similar patterns, *e.g.*, disulfide bridges, in addition to some particular features [6,17,21,29-31].

PnTx1 and its recombinant toxin (rPnTx1) inhibit different subtypes of voltage-dependent sodium channels (Nav), but not the cardiac isoform Nav1.5 [32,33]. These toxins have a particular high affinity for the neuronal isoform Nav1.2. Both PnTx1 and rPnTx1 are promising for the development of new drugs, considering the reports on their specificity and selectivity.

PhTx2 toxins inhibit sodium channel inactivation. These toxins are the main responsible for the strongest neurotoxic effects of venom [17,18,23,34]. Two of them - PnTx2-5 and PnTx2-6 toxins - are responsible for the penile erection observed in the envenomation caused by *Phoneutria* [29], an effect associated with the nitric oxide (NO)/cyclic GMP pathway. These toxins have been explored as models for the development of a potential drug for the treatment of erectile dysfunction [34-36].

The PhTx3 family contains the most heterogeneous toxins. This group includes PnTx3-1, a blocker of inactivating potassium channels currents associated with Ca^2+^ oscillations and pacemaker activity [37]. In contrast, the other toxins of this family (PnTx3-2, PnTx3-3, PnTx3-4, PnTx3-5 and PnTx3-6) block voltage-gated calcium channels with different activities/affinities [38,39]. PnTx3-3 and PnTx3-4 inhibit calcium influx [40], exocytosis, and glutamate release in synaptosomes [41,42]. Besides, it has been shown that PnTx3-6 and Tx3-5 can induce analgesic effects and are efficient in the treatment of persistent pathological pain [43-47].

PhTx4 family of *P. nigriventer* neurotoxins, comprises toxins with insecticidal activities that modulate insect sodium channels [31,48,49]. Despite this apparent lack of toxicity to mammals, PnTx4(5-5) was shown to inhibit NMDA ionotropic glutamate receptors in rat brain neurons [50]. Like PnTx(3-6 and 3-5), PnTx4(5-5) and PnTx4(6-1) showed analgesic effects in different pain models [51,52].

Using transcriptomic and proteomic approaches Diniz et al. [53] revealed the presence of novel compounds in *P. nigriventer* venom. It was found that cysteine-rich peptide toxins are the most abundant components displaying a conserved disulfide scaffold. Other components are proteinase inhibitors, metalloproteinases and hyaluronidases [53]. All these studies have shown that *P. nigriventer* venom has been intensively investigated for drug discovery and their toxins have shown a great biotechnological potential associated with their biochemical and pharmacological properties, but only few studies have focused on the insecticidal properties of the venom components [49]. Since the *Phoneutria* venom is not completely explored, the study of this venom has gained considerable interest and prominence in the scientific community. However, the venoms of other *Phoneutria* species remain poorly investigated.

Paiva et al. [54] carried out the transcriptomic analysis of the spider *Phoneutria pertyi* venom glands and revealed a high similarity to toxins described for the *Phoneutria* genus. In order to gain further information on venoms of other species from the *Phoneutria* genus and assess their biotechnological potential, in this work, we conducted a comparative study on the venoms of *P. nigriventer*, *P. eickstedtae* and *P. pertyi*. 

## Methods

### Animals and venoms

*P. nigriventer* (Pn) and *P. pertyi* (Pp) specimens were collected in Minas Gerais State (MG), and *P. eickstedtae* (Pe) in Goiás State, Brazil. The collection was carried out according to the national license for collection provided by the Brazilian Biodiversity Information and Authorization System (SISBIO 21102-7 2015-2016), whereas the obtained License for Access to Genetic Patrimony (Sisgen A55CA3C) provides access to genetic heritage for conservation and sustainable use of biodiversity. 

Spiders were kept in the Scientific Arachnidarium at Ezequiel Dias Foundation (MG) and the venoms obtained by electrical stimulation of fangs, as described by Barrio and Vital Brazil [55], with modifications [21]. Venoms were homogenized in ultrapure water at 4 ºC, centrifuged (20 min; at 20,000 x g; at 4 ºC) and the supernatants collected, lyophilized, and stored at -20 ºC. Venom samples were labeled according to species, as Pn-V, Pp-V and Pe-V. The protein concentration was determined by the BCA method developed by Smith et al. [56].

### Enzymatic assays

*Zymography*

Gelatinolytic and hyaluronidase activities were assayed by zymography. *Phoneutria* venoms samples (50 μg) were subjected to SDS-PAGE in 12.5% gels containing either 0.1% gelatin or 1.5% hyaluronic acid copolymerized under non-reducing conditions, according to Heussen and Dowdle [57] and Gouveia et al. [58]. After electrophoresis, the gels were washed in 2.5% (v/v) Triton X-100 solution for 1 h to remove SDS and rinsed in water to remove Triton X-100. 

For the gelatinolytic activity assay, gels were incubated in 0.2 M phosphate buffer, pH 8.0, at 37 ºC for 40 min and stained with Coomassie blue R-250. For the hyaluronidase activity assay, the gels were incubated in 0.2 M phosphate buffer, pH 6.0, at 37 ºC for 120 min, washed with water and kept for 30 min in 12.5% (v/v) trichloroacetic acid at 37 ºC. The gels were then washed six times with 3% (v/v) acetic acid solution and incubated in the dark with 1% (w/v) periodic acid for 1 h at 25 ºC. Next, the gels were rinsed with water and kept in 0.5% (w/v) potassium metabisulfite for 30 min at 25 ºC. Finally, the gels were washed with water and stained with 0.5% (v/v) Alcian Blue for 4 h. Gelatinolytic and hyaluronidase activities were revealed by colorless bands in the gels.

*Colorimetric and turbidimetric assays*

Proteolytic activity of the venoms was quantified using dimethyl-casein (DC) as substrate, according to Lin et al. [59] with adaptations. Assays were carried out in PBS buffer, pH 8.5 and 50 μL of DC 0.2% in a final volume of 100 µL. The reactions were initiated by the addition of 5 µg of crude venom, followed by incubation at 37 ºC for 30 min, and stopped by boiling in a water bath (3 min). Subsequently, 50µL trinitrobenzenesulfonic acid (TNBS) (0.1%) and 50 µL sodium bicarbonate buffer (4%) were added and the mixture kept at 50 ºC for 30 min. Next, 50 µL SDS (10%) and 25 µL 1 M HCl were added to the mixture. The quantification of hydrolyzed substrate was determined by absorbance at 340 nm. Proteolysis was expressed as Acquired Colorimetric Unit (ACU). One ACU equals to a gain of 1 absorbance unit at 340 nm. Trypsin was used as positive control, while negative controls comprised the absence of the enzyme or venoms.

Hyaluronidase activity was assayed by turbidimetric method using hyaluronic acid (HA) as substrate, according to Di Ferrante [60]. The assay mixture contained 28 µL of HA (0.1 µg/µL) from human umbilical cord (Sigma-Aldrich), 0.2 M sodium acetate buffer, pH 5.6 (28 μL) and 5 µg of venoms samples in a final volume of 340 µL. Mixtures were incubated at 37 ºC for 15 min and the reaction was stopped by adding 28 µL of hexadecyltrimethylammonium bromide (CTAB) in 2% NaOH. The resulting turbidity was read at 400 nm and the activity expressed as percentage of hydrolyzed hyaluronic acid, considering that the negative control showed 0% hydrolysis, or 100% hyaluronic acid present in the sample. Differences in enzymatic activities between venoms were analyzed using the GraphPad Prism V8.0 software by ANOVA two way (p < 0.01).

### Electrophoresis

In order to compare the protein profiles of the venoms, samples (100 μg) of Pn-V, Pp-V, and Pe-V were submitted to electrophoresis (Tricine-SDS-PAGE), according to Schägger and Jagow [61], under non-reducing and reducing conditions. 

Electrophoresis was performed in a discontinuous vertical system with 15%, 10%, and 4% gels for separating, spacing, and stacking, respectively. Electrophoresis took place overnight at 4 ºC, running at 30 mA in a 0.1 M tricine running buffer, pH 8.5. Gels were stained overnight with 0.05% Coomassie Blue G 250, and images were recorded with an Image Scanner III (GE Healthcare).

### Fractionation of *Phoneutria* venoms and purification of toxins

Fractionation of *Phoneutria* venoms (Pn-V, Pp-V, and Pe-V) and purification of toxins were carried out according to Richardson et al. [21]. Briefly, lyophilized venom samples (30 mg) were dissolved in 2 mL of aqueous 0.1% TFA (solution A) and centrifuged at 5700 x g for 10 min at 4 ºC. The supernatant was applied onto a preparative Vydac C4 column (2.2 x 25 cm) equilibrated with solution A and eluted with a segmented gradient of solution B (100% acetonitrile containing 0.1% TFA), at a flow rate of 5 mL/min (HPLC). Peptides or proteins were detected at 214 nm. Fractions were manually collected, lyophilized, and stored at -20 ºC until use.

For the purification process (toxin isolation), two fractions were selected based on similar elution/retention times to those from previous purifications of *P. nigriventer* venom shown to contain the toxins Tx1 and Tx2-6 [21]. These fractions were processed according to Richardson et al. [21]. Initially, the fractions were reconstituted in 10 mM sodium phosphate buffer, pH 4.7 (solution A) and submitted to cation-exchange-HPLC on a GE Hitrap SP column (0.7 cm x 2.5 cm). The column was equilibrated with solution A and eluted using a linear gradient of 0 to 100% of 10 mM sodium phosphate buffer containing 0.5 M NaCl, pH 4.7 (solution B) at a flow rate of 1 mL/min.

The main protein components from ion-exchange chromatography were further desalted in an analytical RP-HPLC column (218TP54 small pore, 250 mm x 4.6 mm, Vydac, Grace, Columbia, MD) equilibrated with aqueous 0.1% TFA. Fractions were eluted with a gradient of 0 to 70% of solution B (0.1% TFA/ACN) at a flow rate of 1.0 mL/min. 

### Mass spectrometry analysis

*MALDI-TOF analysis*

Molecular masses of components of preparative RP-HPLC fractions were determined by MALDI-TOF MS using a Bruker Autoflex III Smartbeam instrument (Bruker Daltonics, Billerica, MA, USA). External calibration procedures were performed using commercially available standard protein mixtures (Bruker Daltonics, Billerica, MA, USA), according to different mass range: Protein Standard II (10 kDa - 70 kDa); Protein Calibration Standard I (4 kDa - 20 kDa) and Peptide Calibration Standard II (700 Da-3.5 kDa). Prior to analysis, crude venoms were submitted to ZipTip C4 pipette tips (Millipore Co., Burlington, MA, USA) and eluted with 50% (v/v) aqueous ACN containing 0.1% (v/v) TFA. Salt-free samples were lyophilized and solubilized in 0.1% (v/v) aqueous TFA. Next, samples (0.5 µL) were spotted on anchorchip target plates and mixed with 0.5 µL matrix solution. Three different matrices were used, α-cyano-4-hydroxycinnamic acid (HCCA), sinapinic acid (SA) and 2,5-dihydroxybenzoic acid (DHB) matrices that were prepared according to the manufacturer. Briefly, matrices (10 mg/mL) were prepared solved in aqueous ACN solution (1:2) containing 0.1% TFA. The percentage of ACN was used according to each matrix (HCCA- 70%), (SA-40%) and DHB (20%). Analyses were performed in different mass ranges (2000 - 125000). MS spectra were acquired under positive linear and/or reflector ion modes using FlexControl software (Bruker Daltonics). The laser power (355 nm) was tuned manually for an optimal signal. Data were processed and analyzed using FlexAnalysis (Bruker Daltonics) and graphics built with SigmaPlot (Systat Software, Inc) and Microsoft Excel. Besides, MALDI/TOF also was used to confirm the purified toxins (Tx1 and Tx2-6) in positive/linear mode (mass range 2-20000 (m/z) using sinapinic acid as matrix.

In addition, strict criteria were adopted to consider the mass values, of which: (i) the series of the monoisotopic chain or the isolated peak should be conspicuous; (ii) the difference between peaks of the same monoisotopic series should be 1 Da; (iii) intensity of the components was > 5.0 x 10^3^ and (iv) less intense peaks were excluded, for their mass differences could indicate common events in the experiment, such as methylations, oxidations, among others.

*Amino acid sequence*

Purified toxins were subjected of their N-terminus by employing the automated Edman degradation sequencing technique, using a Shimadzu PPSQ-21A (Tokyo, Japan). Subsequently, toxins were subjected to an *in-solution* digestion protocol was used as described by Sanson et al. [62]. Briefly, samples containing 50 µg protein were solubilized in 25 mM ammonium bicarbonate (AMBIC) followed by addition of RapiGest SF Surfactant (Waters, UK) to a final concentration of 0.06% (w/v), and heated at 80 ºC for 10 min. Subsequently, the samples were reduced using 3.3 mM dithiotreitol (DTT) for 10 min at 60 ºC and alkylated with 9.4 mM iodoacetamide (IAA) at room temperature for 45 min, in the dark. MS-grade trypsin (Promega) was added at an enzyme/substrate ratio of 1:50 and digestion proceeded at 37 ºC overnight. Trypsin inactivation and surfactant precipitation were achieved by addition of 0.5% TFA followed by incubation for 40 min at 37 ºC. Finally, the samples were centrifuged for 15 min at 20000 x g at 7 ºC, and the supernatants were stored at -20 ºC until use.

Samples (2 µL) of the supernatants of each trypsinized toxin were subjected to liquid chromatography coupled to mass spectrometry (LC-MS /MS) analyses in a nanoUHPLC UltiMate® 3,000 (Dionex, San Jose, USA) interfaced with a Q Exactive mass spectrometer (Thermo Scientific®, Bremen, Germany), using a Nanospray Flex Ion source and a nano-bore stainless steel emitter (150 μm OD 30 μm ID, Proxeon, Thermo Scientific, Bremen, Germany). A peptide clean-up step was first achieved using a Nano-Trap Acclaim PepMap100 C18 column (100 μm × 2 cm, 5 μm, 100 Å, Thermo Scientific, Whaltham, MA, USA), followed by sequential elution peptides bound to EASY-Column ™ Capillary C18 Columns (75 μm × 10 cm, 3 μm, 120 Å, Thermo Scientific) using an acetonitrile gradient. Formic acid at 0.1% (solution A) and 80% acetonitrile plus 0.1% formic acid (solution B) were used as eluents. The column was equilibrated for 3 min with solution A at a flow rate of 0.3 μL/min. Elution proceeded with 3.8% solution B and gradually increased to 99% in 152 min, remaining so for another 10 min. Mass spectrometric analysis was performed on positive mode under 3.45 kV with interface temperature kept at 250 ºC. Spectra were acquired with a MS1 resolution of 70,000, maximum injection time of 120 ms, AGC target 1 × 10^6^, scan range 300-2000 *m/z*, and charge states ≥ +2 and ≤ +5. Peptide fragmentation of up to 12 most intense ions was carried out by high energy collisional dissociation. MS/MS spectra were obtained under 35,000 resolution, isolation window 2 *m/z*, normalized collision energy of 30 V, and dynamic exclusion time of 40 s.

The peptides were identified using the PEAKS Studio 8.5 program (Bioinformatics Solutions Inc, Canada) through mass spectral data interrogation searching against a compilation of toxin sequences from animals’ venoms deposited in NCBI database (185,632 sequence) (NCBI- https://www.ncbi.nlm.nih.gov/) and all sequences in ArachnoServer (1,500 sequence) (http://www.arachnoserver.org/) (187,132 sequences total, downloaded on 16 November 2020). The following search parameters were considered: up to 2 missed trypsin cleavage sites were accepted; a precursor error of 10 ppm; and product error of 0.1 Da. Carbamidomethylation of cysteine residues (+57.02 Da) and methionine oxidation (+15.99 Da) were set as fixed and variable modifications, respectively. Multiple sequence alignment was performed by the Clustal Omega/EMBL-EBI (https://www.ebi.ac.uk/Tools/msa/clustalo/).

### Whole-cell patch clamp

Toxin-1 isoforms (PnTx1, PpTx1 and Pe-Tx1) isolated from the venoms of different *Phoneutria* species were submitted to electrophysiological assays in order to verify their action on sodium channels. The experiments were carried out at room temperature (20 - 22 ºC ) in whole-cell patch-clamp mode with a HEKA amplifier (EPC10 USB, Germany), using HEK293 cells held at -80 mV. The P/4 protocol was used to subtract capacitance and linear leak currents. Patch pipettes resistance varied between 1.2 - 2.5 MΩ. During the test pulse, the membrane was depolarized to 0 mV for 50 ms, following a pre-pulse to -100 mV for 100 ms, every 5 seconds.

HEK293 cells permanently expressing Na_v_1.6 channels were incubated at 37 ºC in 5% CO_2_, in high glucose DMEM supplemented with 10% fetal bovine serum and 1% glutamax. Cells were split once a week using trypsin-EDTA solution (0.05%, Gibco), and the experiments carried out 24 - 48 h afterwards. The external solution used to acquire the data was: 130 mM NaCl, 5.4 mM KOH, 1.8 mM CaCl_2_, 1 mM MgCl_2_, 10 mM glucose, 10 mM HEPES, pH 7.4. Pipette solution composition was: 100 mM CsF, 10 mM NaCl, 5 mM MgCl_2_, 11 mM EGTA, 10 mM TEA-Cl, 10 mM HEPES, pH 7.2. 

Toxin-1 from the venoms of Pn-V (PnTx1, 1 µM), Pp-V (PpTx1, 3.25 µM), and Pe-V (PeTx1, 1 µM) were diluted in external solution and delivered (flow 160 - 200 µL/min) by means of a perfusion system (VM4, ALA Scientific Instruments) with the outlet placed near the cell. Bovine serum albumin (0.1%; Sigma-Aldrich, ref. 7030) was added to the toxin solutions to avoid adsorption. Current recovery was measured 350 s after the beginning of washout.

## Results

### Enzymatic activity of *Phoneutria* venoms

**Figure 1.**
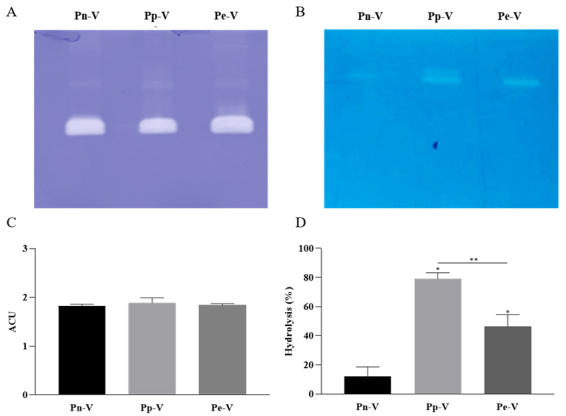
Enzymatic activities of *P. nigriventer* (Pn-V)*, P. pertyi* (Pp-V) *and P. eickstedtae* (Pe-V) venoms. Zymography of crude venoms (50 µg) in **(A)** 12.5% SDS-PAGE 0.1% gelatin and **(B)** 12.5% SDS-PAGE 1.5% HA. Enzymatic activities measured by spectrophotometry with **(C)** dimethyl-casein and **(D)** hyaluronic acid as substrates. Data are presented as mean ± SEM; p < 0.01 represented with (*) for Pp-V/Pe-V *versus* Pn-V and with (**) for Pp-V *versus* Pe-V.

Analysis of the gelatinolytic and hyaluronidase activities of Pn-V, Pp-V, and Pe-V were carried out through zymography using gelatin and hyaluronic acid as substrates. All three venoms exhibited prominent gelatinolytic and hyaluronidase activities, evidenced by the large colorless bands in the gels (Figure 1A and 1B). These bands presented similar migration in both zymograms. A similar degradation pattern was observed for the gelatinolytic activity of all venoms. In contrast, the hyaluronidase activity of Pp-V was higher compared to the other two venoms. To quantify and compare proteolytic and hyaluronidase activities from the crude venoms, we performed a colorimetric test on dimethyl casein and turbidimetric assays, respectively (Figure 1C and 1D). The results corroborated the zymography data and showed similar specific activity for all the venoms concerning dimethyl casein activity (Figure 1C), and some differences for that of hyaluronidase. Pp-V and Pe-V degraded HA 6.5 and 3.8 times more efficiently than Pn-V, respectively (Figure 1D). 

### Protein profile of *Phoneutria* venoms

**Figure 2.**
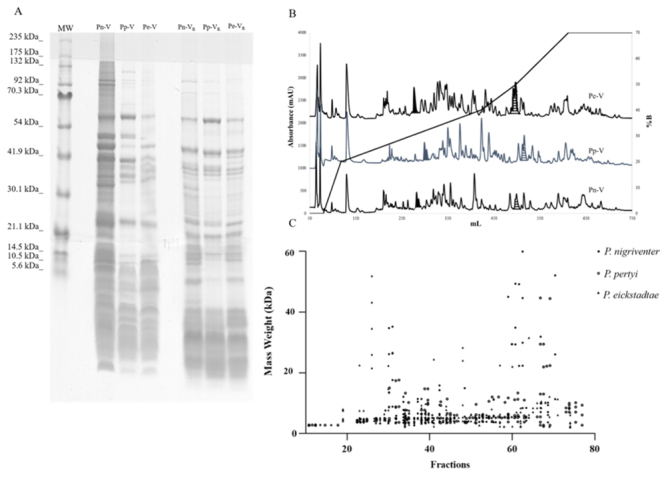
Comparison of the protein profiles of the crude venoms of *P. nigriventer* (Pn-V), *P. pertyi* (Pp-V) and *P. eickstedtae* (Pe-V). **(A)** SDS-PAGE (discontinuous vertical system with 15%, 10%, and 4% for separating, spacing, and stacking gels, respectively - 100 µg of each venom), under non-reducing (left) and reducing (β-mercaptoetanol 4%, v/v) (right) conditions. The positions of molecular mass markers are indicated on the left. The gel was stained with Coomassie brilliant blue. **(B)** RP-HPLC profile of crude venoms (30 mg) on preparative Vydac C4 column (2.2 cm x 25 cm). The column was equilibrated using solution A [0.1% TFA (v/v) in ultrapure water] and eluted using solution B [0.1% TFA (v/v) in acetonitrile]. Protein elution was monitored by absorbance at 214 nm. Hachured and striped peaks indicate the fractions corresponding to Tx1 and Tx2-6, respectively. **(C)** Mass distribution of the fractions from preparative RP-HPLC.

The protein profiles of the crude venoms from the three species were compared through SDS-PAGE. The analysis revealed that the venoms appear to be similar regarding the number of components (~40 protein bands are visualized) and the pattern of mass distribution (2 - 175 kDa), with the majority migrating in the mass range below 6.0 kDa. However, there are visible differences in the intensity of some components in these venoms (Figure 2A). 

In addition, we compared preparative reversed-phase HPLC profiles of the venoms, using an extended gradient of acetonitrile in 0.1% aqueous TFA. The profiles obtained were quite similar, with venoms fractionating into 70 main protein components eluted mostly between ~20 - 55% acetonitrile (Figure 2B). These profiles revealed peaks with elution times and features matching the toxins PnTx1 and PnTx2-6 previously isolated from the *P. nigriventer* venom [21], which were eluted with roughly 30 and 50% acetonitrile, respectively (Figure 2B). 

Furthermore, the similarity between these *Phoneutria* venoms was corroborated by the comparison between the masses of the fractions obtained by preparative RP-HPLC, identified by MALDI-TOF (Figure 2C). In accordance with SDS-PAGE profiles, these results show that low molecular weight proteins (≤10 kDa) represent the largest proportion of the components of the venoms, although some molecules are distributed throughout the high molecular mass range.

### Toxin isolation and chemical characterization

**Figure 3.**
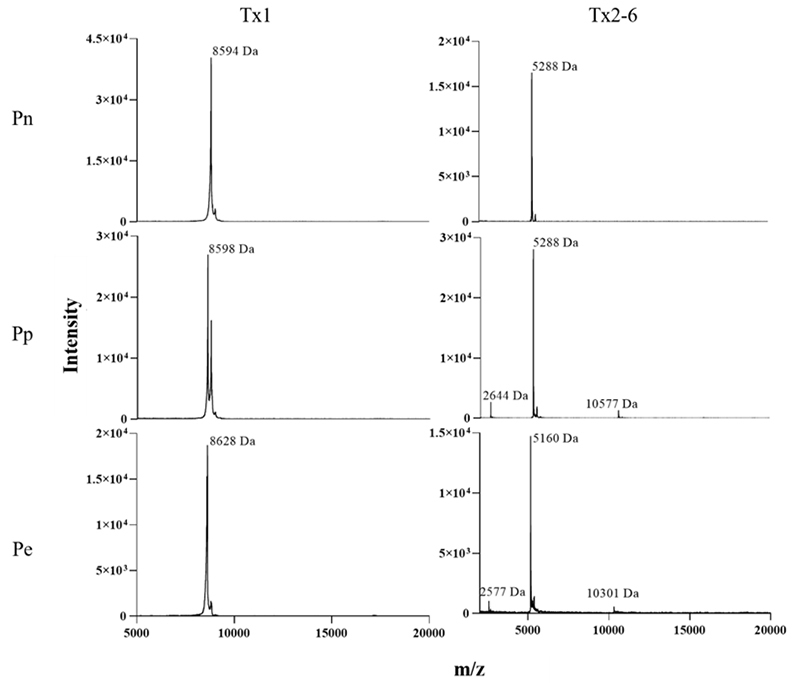
Chemical analyses of purified toxins. MS spectra obtained, respectively, for toxins exhibiting dimer formation and double charge on MALDI/TOF in positive/linear mode - mass range 2-20000 (*m/*z) using sinapinic acid as matrix.

In order to ascertain the presence of Tx1 (PnTx1, PpTx1 and PeTx1) and Tx2-6 (PnTx2-6, PpTx2-6 and PeTx2-6) isoforms, fractions from preparative RP chromatography with elution times corresponding to these toxins (Figure 2B) were subsequently fractionated on cation-exchange-HPLC. The major protein peak obtained in this chromatography was eluted as a symmetrical peak in RP-HPLC (data not shown). Mass spectrometry analysis of these samples on a MALDI/TOF instrument revealed a high degree of homogeneity. In Pn-V, Pp-V, and Pe-V signals were evidenced at *m/z* values of 8594, 8598 and 8628 Da, there were also signals of 5288, 5288, and 5160 Da, for Pn-V, Pp-V, and Pe-V, respectively, all values described for Tx2-6 toxins (Figure 3).

**Figure 4.**
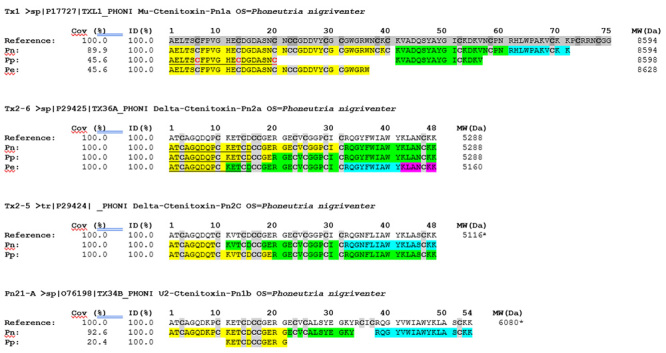
Alignment of the partial amino acid sequence of toxins identified. Cysteines (Cys, C) residues are highlighted in gray color. Reference: Sequence, accession code and name of *P. nigriventer*’s toxin deposited in Uniprot database. The size of identified peptides are highlighted in yellow, green, blue and pink. MW: molecular weight (Da). *Theoretical mass value obtained from PeptideMass (www.expasy.com). Mass value according to Richardson et al. [21]. Underlined sequences were obtained by Edman Degradation method. Positions of cysteine residues (labeled in red) were inferred by homology.

In order to confirm the toxin sequences, the N-terminal were determined by Edman degradation. The Tx1 isoforms (PnTx1, PpTx1 and PeTx1) were obtained 20 amino acids residues, while to Tx2-6 isoforms (PnTx2-6, PpTx2-6 and PeTx2-6), 15 amino acids residues (Figure 4). Besides, amino acid sequences of tryptic fragments were obtained by database interrogation of mass spectral data (Figure 4). Using the venom sequences database from NCBI and spider toxins from ArachnoServer, we found that the peptide fragments showed identity to PnTx1 (P17727-1) and PnTx2-6 (P29425). In addition, the purified fractions of Pn-V and Pp-V also present peptides were identified from PnTx2-5 (P29424) and Pn21-A (O76198) isoforms (Figure 4).

Sequence coverage percentage obtained for PnTx1, PpTx1 and PeTx1 were 89.9%, 45.6% and 45.6%, respectively, while for all Tx2-6 and two Tx2-5 isoforms obtained were 100%. On the other hand, the coverage of Pn21-A and Pp21-A were 92.6% and 20.4%, respectively. Already identity was 100% for all peptides obtained in that work (Figure 4). Therefore, Pp-V and Pe-V contain isoforms of the Tx1 and Tx2-6, moreover, in Pp-V was possible to still find PpTx2-5 Pn21-A isoform. 

### Effect of *Phoneutria* toxins on Nav1.6 sodium channels

**Figure 5.**
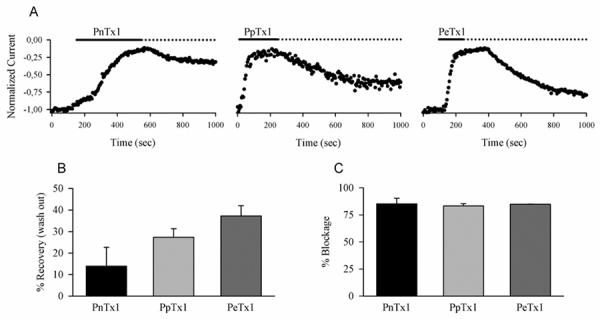
Different Tx1 isoforms block Nav1.6 sodium current reversibly. **(A)** Time course of the blockage of the sodium current peak by Tx1 isoforms from different *Phoneutria* species and washout kinetics. **(B)** Recovery of sodium current blockage after 350 s of washout. **(C)** Percentage of Nav1.6 sodium currents blockage by Tx1 isoforms after stabilization.

 To verify whether different isoforms of the Tx1 toxin would exhibit the same effect, we compared their ability at blocking Na_v_1.6 sodium channels (Figure 5). Toxins were tested at concentrations of 1μM for PnTx1 and PeTx1 and 3.25 μM for PpTx1. Although PpTx1 concentration was slightly higher, the effect was already supramaximal and had no influence in the maximum blockage. All toxins PnTx1, PpTx1 and PeTx1 were able to block the channels however the kinetics of blockage and recovery washout were different among the isoforms. PnTx1 had the slowest effect, suggesting that some amino acids that compose its binding site might be protected inside the channel structure, but once they are exposed, the toxin bind to the channel and it was hard to wash it out (Figure 5A). On the other hand, PpTx1 and PeTx1 did not appear to bind to the channels (on-rate) in a voltage-dependent way, with maximum inhibition being reached after few pulses (Figure 5A). At 350 s, 27.4 ± 3.91% and 37.25 ± 4.77% of the current was recovered after PpTx1 and PeTx1 washout, respectively (Figure 5B). Once these toxin isoforms were bound to the channels, the off-rate was slow and the recovery after washout incomplete, at least for the duration of the experiment (Figure 5B). The blockage was 85% for all of them (Figure 5C), even for PpTx1 at a higher concentration, suggesting that saturation had been reached.

## Discussion

Animal venoms contain toxic components that induce different physio-pharmacological effects by acting on molecular targets, showing immense biotechnological potential. Some toxins that act on different types of ion channels, with the considerable advantage of binding selectively, have been identified in the venom of the spider *P. nigriventer*[17]. Possibilities for the development of new drugs based on these toxins, as well as their use for a deeper understanding of complex physiological processes, have been described[35,36,50,51,63].

In spite of the potential pharmaceutical benefits offered by *P. nigriventer* toxins, there is limited information on venoms of the same genus, and their toxins remain poorly characterized. In order to obtain more information on toxins of other species from the *Phoneutria* genus and assess their biotechnological potential, we proposed the present study. Through enzymatic activities, SDS-PAGE, HPLC profiles, and mass spectrometry analysis, we conducted a comparative investigation on the protein profiles of crude venoms of three different spiders of the genus *Phoneutria*: *P nigriventer, P. eickstadae*, and *P. pertyi*, correlating them with biological activities. In addition, two molecules of Pn-V toxins were purified, one of which had its action on sodium channels analyzed. 

As proteolytic and hyaluronidase activities are common in animal venoms, we analyzed the gelatinolytic and hyaluronidase activities of *Phoneutria* venoms. We observed that these venoms exhibited gelatinolytic activity with a similar degradation pattern, whereas hyaluronidase activity appears to be more potent in Pp-V and Pe-V compared to Pn-V.

Venom proteases are multifunctional compounds, being related from allergic reactions and blood coagulation to toxin processing. Proteases have already been identified in several spider venoms, *e.g.*, a protease from the venom of *Loxosceles* sp. genus is associated with injury to blood vessels, impaired endothelial cell adhesion, and cell death induced by this venom [64,65]. Serine proteases were described in *P. nigriventer, P. reidyi,* and *P. keyserlingi* venoms, being associated with post-translational modification of venom components [21]. Serine proteases (*e.g.*, allergen), metalloproteinases (*e.g.*, astacin, zinc metalloproteinase, and neprilysin), and cysteine-rich secretory protein (CRISP) were found in the *Grammostola iheringi* spider venom [8]. 

The presence of hyaluronidases has also been identified in the venoms of various animals such as reptiles and arthropods, as well as from other sources, such as fungi and bacteria, mammalian organs, and corporal fluids [66]. These enzymes hydrolyze the glycosaminoglycan that form the extracellular matrix, having hyaluronic acid (HA) as their preferred substrate. In animal venoms, these glycosidases are thought to increase plasma membrane absorption and reduce viscosity, ensuring that the toxic molecules can reach their targets in the victim’s organism, consequently contributing to the overall toxicity of these venoms. 

Therefore, hyaluronidases act as spreading factors for the toxins during envenomation. The enzymatic action of hyaluronidases is also associated with several pathophysiological processes, as fertilization, embryogenesis, angiogenesis, wound healing, sepsis, metastasis, among others [67]. Due to these effects, hyaluronidases show great biotechnological potential, having been used for clinical applications, *e.g.*, to reduce cancer progression and in ophthalmological procedures [66].

Comparison of the SDS-PAGE and RP-HPLC profiles of venoms of the three *Phoneutria* species showed similarity in the overall venom composition. When these venoms were subjected to separation by electrophoresis, a high number of similar proteins that migrated as protein bands with apparent molecular weights ranging from 2 - 175 kDa, under reducing and non-reducing conditions, was revealed (Figure 2A). As expected, most components displayed molecular weights lower than 6 kDa (Figure 2A), corresponding to neurotoxins commonly found in arthropod venoms. In addition, SDS-PAGE showed less complexity under non-reducing conditions than is typical under reducing conditions (range 15 - 40 kDa), indicating the presence of proteins with more than one polypeptide chain and linked by disulfide bonds, or protein complexes.

Richardson and cols. demonstrated that when *P. nigriventer, P. keserling* and *P. reidyi* venoms were subjected to 2-D electrophoresis, approximately 80 spots were visible in each case [21]. However, mass fingerprinting by RP-HPLC/mass spectrometry analysis revealed that each venom contains over 150 different molecules [68]. In the present work, 142, 149, and 214 mass values (> 2 kDa) were detected for Pn-V, Pp-V, and Pe-V, respectively, when each fraction of these venoms from preparative RP-HPLC were subjected to MALDI/TOF analysis*.* Using a similar approach, Escoubas et al. [69] found 633 peptides components in the venom from *Atrax robustus* spider (male) and 1018 in the *Hadronyche versuta* (female). While Duran et al. [70] recorded 83 components in *Hadronyche valida* venom. Li et al. [19] compared the venom from two fishing spiders of *Dolomedes* genus, for *D. mizhoanus* were described 302 components, while for *D.sulfurous*, they found 378 peptides [19,69,70]. 

Therefore, our data suggest that the venoms studied show general similarity in their components, as well as minor qualitative and quantitative differences between them. The venoms of *Phoneutria* species exhibited the overall common features: (i) number of highly similar protein peaks (on average 80) were eluted with comparable elution/retention times in preparative RP-HPLC; (ii) electrophoretic profiles and mass scatter plot analysis showed a predominance of low molecular mass molecules (~6.0 kDa); (iii) multiple masses per fraction and (iv) several fractions containing masses with similar values that were co-eluted simultaneously, suggesting the presence of many isoforms. 

Intraspecific differences in animal venom composition are frequently noticed and several factors have been shown to influence it, such as food availability, season, environment, stages of maturation, and sex of each specimen [20,21]. In addition, the post-translation process (proteolysis and C-terminal alterations) is a recurrent source for this intraspecific variability among venoms [68,71]. Besides, the variability among venoms of species from the same genus has also been described. This interspecific variability results in isoforms of the same toxin, with strong similarities in their structures and biological activities [21,71]. Furthermore, according to the literature, this venom variation can be highly useful as a marker to identify species [72-74]. Despite of some studies have reported that amino acid substitution alters the functional activity, it is not clear if in our approach we have the effects of these modifications, such as evolutionary implications. Further studies will be necessary to confirm this.

Considering the existence of intraspecific differences and the variability among venoms of species from the genus *Phoneutria*, in this study the samples of venoms were prepared from animals of both sexes, sent to Ezequiel Dias Foundation Aracnidarium by the population or collected from different habitats. To confirm the presence of the isoforms and the functional similarity of the toxins from venoms of different species, two fractions from preparative RP- HPLC, eluted with ~30 and 50% acetonitrile (Figure 2B), which correspond to the toxins PnTx1 and PnTx2-6 were fractioned by ion exchange and analytical reversed-phase chromatography. 

After these chromatographies the mass spectrometry analysis was carried out (MALDI/TOF and electrospray) of purified toxins revealed the presence of Tx1 and Tx2-6 isoforms for all three species of *Phoneutria* studied. We found masses of 8594, 8598, and 8628 Da for PhTx1 isoforms, while for PhTx2-6 isoforms the mass values were 5288, 5288, and 5160 Da for Pn-V, Pp-V, and Pe-V, respectively, using MALDI/TOF. As far as we know the literature does not provide explanation as to why the theoretical mass observed for both Tx1 and Tx2-6 (monoisotopic mass = 8663 and 5294) does not match the experimental values obtained (monoisotopic mass = 8594 and 5288) for any of the sequences in the investigated species. For Tx2-6 from *P. eickstedtae* (Pe) the mass difference is even greater (monoisotopic mass = 5160). This experimental mass value was reconfirmed by the use of three distinct matrices for MALDI technique. As shown in Figure 1, we have detected proteolytic activities in the three venom extracts, therefore this mass difference might be attributed to post-translational processing of the respective toxin.

PnTx1 and PnTx2-6 are the best studied toxins from *P. nigriventer.* PnTx1 was the first neurotoxin identified from the venom of *Phoneutria* species [75]. This toxin is composed of 78 amino acid residues (MW 8594.6 Da) cross-linked with 7 disulfide bonds, representing about 0.45% of the whole venom. It is a reversible inhibitor of neuronal sodium channels (Nav1.2/ SCN2A) that binds in proximity to site 1 and displays increasing affinity as the membrane potential is depolarized. PnTx1 induces excitatory symptoms and spastic paralysis in mice [32,76,77]. 

PnTx2-6 (MW 5288 Da) contains 48 amino acids, of which 10 are cysteine residues [29]. This toxin modifies the kinetics of sodium channel inactivation by shifting the voltage dependence of activation towards more hyperpolarized potentials, thus increasing sodium influx [78]. Furthermore, PnTx2-6 was found to induce priapism, representing one the symptoms of envenomation by *P. nigriventer* [21,24]. Due to such effects on penile erection, PnTx2-6 has shown good perspectives for clinical application on erectile dysfunction treatment. PnTx2-6 modulates the nitric oxide (NO)/cyclic GMP pathway, resulting in increased release of NO in the corpus cavernosum tissue, potentially causing penile erection [24,35,36]. Based on the active portion of the native toxin, PnTx2-6 was used as a model to design a 19 amino acid residue peptide, termed PnPP-19, which was able to potentiate erection both *in vivo* and *ex vivo*, in mice. Besides, this synthetic peptide neither showed toxicity, nor affected sodium channels or rat hearts, and also showed low immunogenicity [24,79]. These findings make PnPP-19 a promising molecule, and this compound is under investigation for the development of drugs for the treatment of erectile dysfunction. Since our results showed the PpTx2-6 and PeTx2-6 share 100% identity with PnTx2-6, it is suggested these peptides kept priapism effect and also could be used to drug design. However, it would be necessary more assays to ascertain this hypothesis.

Finally, to verify whether the PhTx1 isoforms are functionally similar to PnTx1, we examined their effect on sodium channels expressed in HEK293 cells. Like PnTx1, PeTx1 and PpTx1 isoforms blocked Nav1.6 currents (Figure 5). However, the kinetics of blockage and washout were quite different among the isoforms. These findings are consistent with small alterations in mass values, suggesting some amino acid substitutions would be responsible for these functional modifications. 

It is already well established in the literature that PnTx2-6 decreases sodium channel inactivation [78]. Although we have not assayed Tx2-6 isoforms from *P. eickstedtae* (Pe-V) and *P. pertyi* (Pp- V), we believe that them has similar effects on sodium channels like PnTx2-6 isoform and similar results would be expected, because these toxins are homologous. The species *P. eickstedtae* and *P. pertyi* are rarer spiders. In addition, since Tx1 is the most abundant toxin, only this molecule was used for electrophysiology assays. We believe that our data, as they are, already bring the news to arouse the curiosity of the scientific community in the area. 

## Conclusion

Our findings have shown that the protein composition and enzymatic activities of Pe-V and Pp-V are highly similar to those of Pn-V, with only subtle differences. Moreover, isoforms of toxins previously described in *P. nigriventer* venom were identified in Pe-V and Pp-V - i.e. isoforms of the toxin Tx1 (PnTx1, PpTx1 and PeTx1) and Tx2-6 PnTx2-6, PpTx2-6 and PeTx2-6), which share high amino acid sequence similarity. Tx1 isoforms were able to block the Nav1.6 channel, though the kinetics of blockage and recovery were different for each isoform. Variations in biological activities and in differences in the venom composition (presence of isoforms as reported in our study) are widely described. In conclusion, these variations are very important for venom diversity as they can enhance the arsenal of biomolecules, thus contributing to the search for animal venom-derived proteins that could be candidate molecules for biotechnological applications.

### Abbreviations

ACN: acetonitrile; ACU: acquired colorimetric unit; AMBIC: ammonium bicarbonate; CaCl_2_: calcium chloride; CHCA: α-cyano-4-hydroxycinnamic acid; CTAB: hexadecyltrimethylammonium bromide; DC: dimethyl-casein; DHB: 2,5-dihydroxybenzoic acid; DMEM: Dulbecco’s Modified Eagle Medium; DTT: dithiotreitol; EDTA: 2-[2-[bis(carboxymethyl)amino]ethyl-(carboxymethyl)amino] acetic acid; EGTA: ethylene glycol-bis-(b-amino-ethyl ether) N,N,N´,N´-tetra-acetic acid; ESI: electrospray ionization; GMP: guanosine monophosphate; HA: hyaluronic acid; HEPES: 4-(2-hydroxyethyl)-1-piperazineethanesulfonic acid; HPLC: high-pressure liquid chromatography; IAA: iodoacetamide; KOH: potassium hydroxide; LC: liquid chromatography; MALDI-TOF: matrix associated laser desorption/ionization-time of flight; MgCl_2_: magnesium chloride; MS: mass spectrometry; MW: mass weight; NaCl: sodium chloride; NaOH: sodium hydroxide; Nav: type voltage-dependent sodium channel; NMDA N-methyl d-aspartate; NO: nitric oxide; Pe: *Phoneutria eickstedtae*; Pe-V: *Phoneutria eickstedtae* venom; Ph: *Phoneutria*; Pn: *Phoneutria nigriventer*; Pn-V: *Phoneutria nigriventer* venom; Pp: *Phoneutria pertyi*; Pp-V: *Phoneutria pertyi* venom; RP: reverse-phase; SDS-PAGE: sodium dodecyl sulfate polyacrylamide gel electrophoresis; TEA-Cl: tetraethylammonium chloride; TFA: trifluoracetic acid; TNBS: 2,4,6-trinitrobenzenesulfonic acid; Tx: toxin; UHPLC: ultra-high-pressure liquid chromatography. 
